# Duplication of superficial femoral artery: imaging findings and literature review

**DOI:** 10.1186/s12880-020-00500-4

**Published:** 2020-08-26

**Authors:** Sung Hyun Yu, Jung Han Hwang, Jeong Ho Kim, Suyoung Park, Ki Hyun Lee, Sang Tae Choi

**Affiliations:** 1 Department of Radiology, Gil Medical Center, Gachon University College of Medicine, 21, Namdong-daero 774beon-gil, Namdong-gu, Incheon, 21565 Republic of Korea; 2 Department of Surgery, Gil Medical Center, Gachon University College of Medicine, 21, Namdong-daero 774beon-gil, Namdong-gu, Incheon, 21565 Republic of Korea

**Keywords:** Anatomic variation, Diagnostic imaging, Lower extremity, Case reports

## Abstract

**Background:**

Duplication of the superficial femoral artery (SFA) is an extremely rare anatomic variation, with few case studies reported. We report one case of the duplicated SFA, discovered by both ultrasonography (US) and computed tomography angiography (CTA). We also reviewed literatures concerning 6 cases of the duplicated SFA (including our present case), and summarized the clinical and imaging features of the anatomic variation.

**Case presentation:**

A 55-year-old woman presented to our hospital with an intermittent cramp in the lateral aspect of the right leg. The patient underwent Doppler US examination on bilateral lower extremity arteries and veins to examine potential vascular abnormality. Incidentally, US discovered the duplicated left SFA and CTA of bilateral lower extremities revealed the anatomic orientation, course, length, diameter and distance of the duplicated left SFA. It was revealed to be divided into two trunks with similar luminal diameter and courses parallel. They reunited at distal thigh level. The findings of US and CTA examination did not correspond with the symptom of the patient, and the patient was discharged.

**Conclusion:**

We report a rare case of the duplicated SFA diagnosed with the combinations of US and CTA examination, which served as valuable imaging methods to detect and diagnose the vascular anatomic variation in lower extremities.

## Background

The precise knowledge of vascular anatomy and its variations is crucial as the treatment with endovascular techniques has increased. Variations of the femoral artery are rarely reported, especially for the superficial femoral artery (SFA) [1–3]. Few cases of the duplicated SFA have been reported with limited combinations of imaging modalities, such as computed tomography angiography (CTA), conventional angiography, ultrasonography (US), or magnetic resonance angiography (MRA) [2, 4–7]. We report a case of the duplicated SFA, which was diagnosed using both CTA and US, with a brief literature review.

## Case presentation

A 55-year-old woman presented to our hospital with nonspecific knee pain in the lateral aspect of the right leg. She had no symptoms on the left leg. She had hypertension, diabetes mellitus, and dyslipidemia, and she was on a treatment for intervertebral disc herniation in another hospital. Her physical examination was nonspecific, and the right and left ankle-brachial pressure index was 1.06 and 0.88, respectively.

**Fig. 1:**
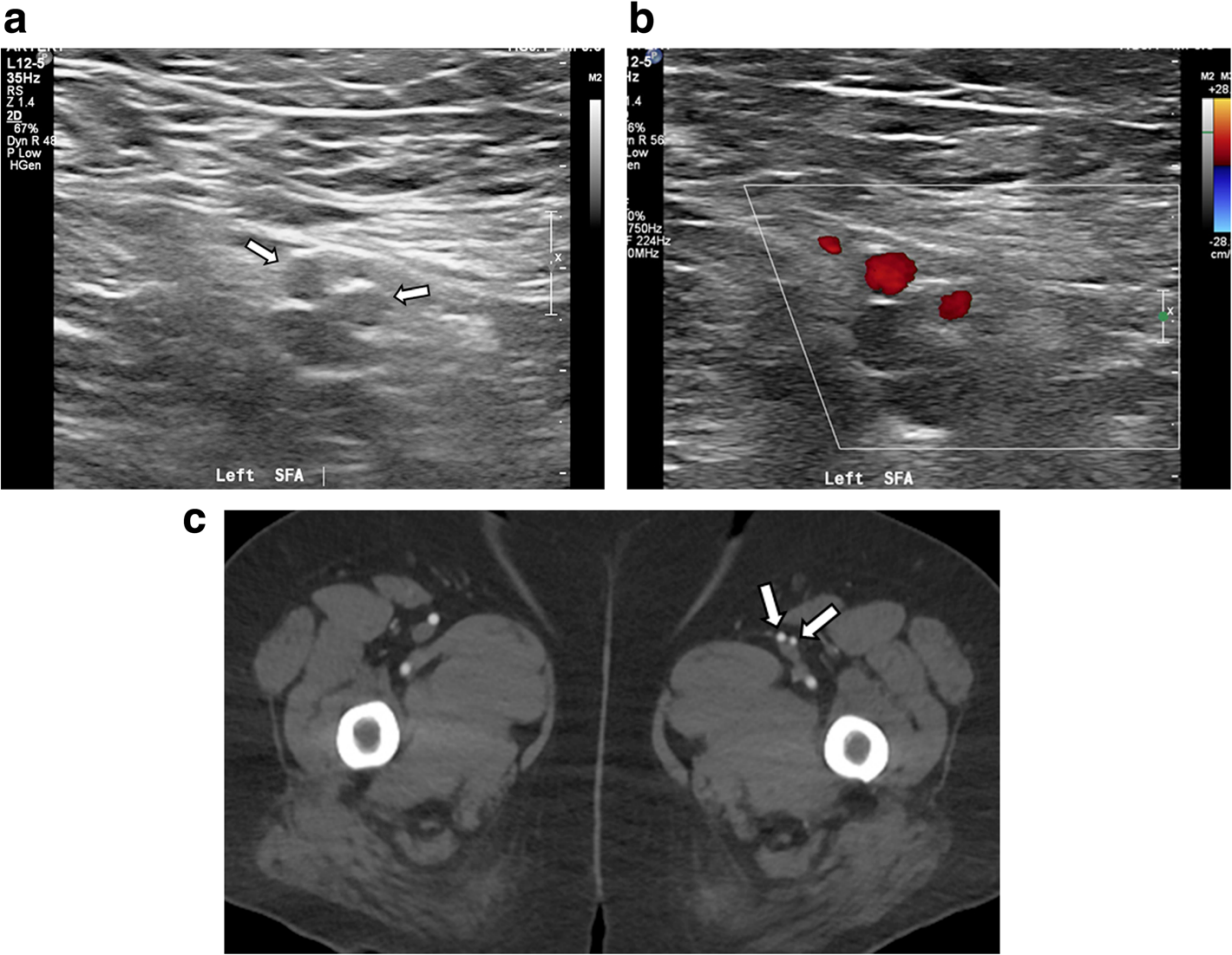
Gray scale (**a**) and color Doppler ultrasonography (**b**) show that the left superficial femoral artery is divided into two trunks (arrows) with similar luminal diameter and courses parallel. The axial image of arterial phase CTA of bilateral lower extremity (**c**) shows the duplicated left superficial femoral artery (arrows). The luminal diameter of the medial one is slightly larger than that of the lateral one. Note normal right superficial femoral artery

The patient underwent Doppler US examination on bilateral lower extremity arteries and veins to examine potential vascular abnormality. There was no abnormality in vessels of the right side. The left SFA was revealed to be divided into two trunks with similar luminal diameter and courses parallel (Fig. 1). They reunited at distal thigh level. No other abnormalities or diseases in vessels of the left side were identified by US examination.

**Fig. 2:**
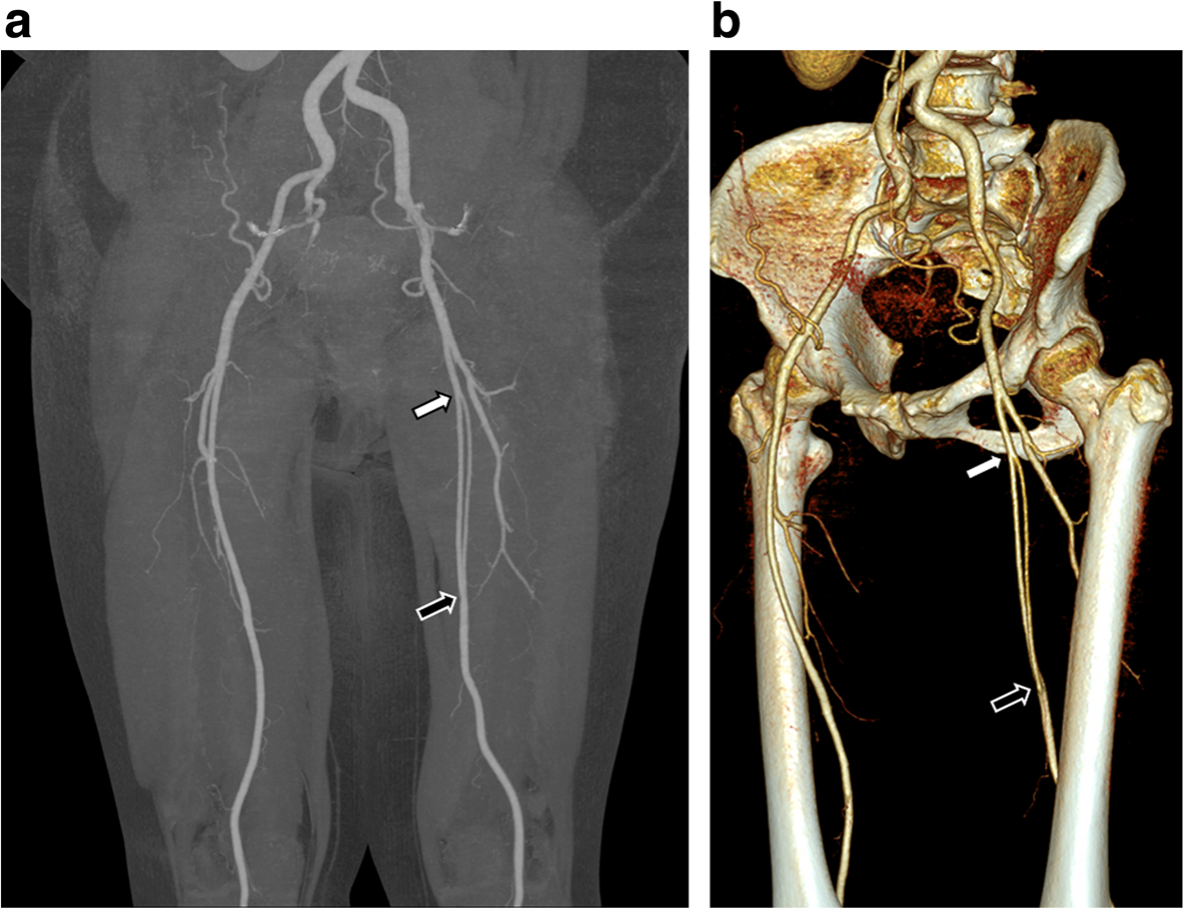
Maximum intensity projection image (**a**) and 3-D volume rendering image (**b**) show the proximal (white arrows) and distal (black arrows) portion of the duplicated left superficial femoral artery

For further evaluation, CTA of bilateral lower extremities was performed. The left SFA appeared to originate from left common femoral artery at the same level of the contralateral side. It appeared to run as a single vessel, 4 cm long, then split into two branches, medial and lateral ones. The luminal diameter of the medial one of the SFA was 5.3 mm, whereas that of lateral one was 4.4 mm, measured in each proximal portion (Fig. 1). Both then traveled 14 cm distal along anterior side of the left superficial femoral vein. They merged at distal thigh level to form single vessel and ran 4 cm distal to enter the adductor hiatus. The anatomic orientation was well visualized in three-dimensional volume rendering and maximum intensity projection images (Fig. 2). There was no evidence of atherosclerotic stenosis or other diseases on the bilateral lower extremity arteries. The findings of US and CTA examination did not correspond with the symptom of the patient, and the patient was discharged.

## Discussion and conclusion

Variations of the femoral artery are rarely reported, including aplasia and hypoplasia of the SFA with associated persistent sciatic artery, duplication or hypoplasia of the deep femoral artery, trifurcation of femoral artery, and duplication of the SFA [1, 2]. To date, only a few cases have been reported on the duplicated SFA with one or two imaging modalities [2, 4–7], and some reports and textbooks discuss embryologic basis for anatomic variant of lower extremities [2, 8–12].

A literature search on PubMed database was performed to review the previous reports concerning the duplicated SFA. The detailed analysis is summarized on Table 1. Including our report, all reported cases of the duplicated SFA have single vessel segments at proximal and distal portions of the duplicated segment. Furthermore, all the reported cases have the duplicated SFA variation in unilateral side of lower extremity [2, 4, 5, 7]. Duplication of SFA occurred in the right side (3 cases) and left side (2 cases), like our case [2, 4–7]. The vascular diameters of each duplicated SFA are equal in the three case reports [2, 4, 5], whereas unequal in two [6, 7]. In our case, the diameter of the duplicated SFA is unequal without atherosclerosis obliterans or other vascular disease. In most of the cases, duplicated SFA were discovered incidentally except two cases with intermittent claudication. Similarly, our patient had a nonspecific knee pain in the lateral aspect of the right leg, but she had no symptoms associated with duplicated SFA. As far as the gender information, previous reports only examined male patients. However, our case described the case of a female patient with duplicated SFA.

**Table 1:** Literature review of reported seven cases of duplicated superficial femoral artery (SFA)

No.	References	Age/Sex	Symptoms & signs	Side	Imaging	Length	Diameter	Ratio	Course	Distance^a^
1	*Kantarci F* et al. [6], 2003	60/M	Claudication	Right	US,MRA	55% ^b^	N/A	Unequal (ASO)	LR	5 cm
2	*Huynh KD* et al. [4], 2010	52/M	Puncture site pseudoaneurysm	Left	CT	38% ^b^	4.2 mm	Equal	LR	23% ^c^
3	*Javerliat I* et al. [5], 2010	80/M	Critical ischemia of lower limb	Right	XA	12 cm	7 mm	Equal	LR	8 cm
4	*Rajadurai VA* et al. [7], 2015	65/M	Peripheral vascular disease	Right	CT,XA	17 cm	5 ~ 7 mm	Unequal (ASO)	LR	4 cm
5	*Hapugoda S* et al. [2], 2016	65/M	Presurgical planning	Left	CT	20 cm	N/A	Equal	LR to AP	4 cm
6	**Our case**	55/F	Intermittent cramp	Left	CT,US	14 cm	5.3 mm/ 4.4 mm	Unequal	LR to AP	4 cm

^a^length between the bifurcation of the SFA and the origin of the duplication

^b^estimated ratio length of the duplicated SFA to the whole length of the SFA

^c^estimated ratio length between the bifurcation of the SFA and the origin of the duplicated SFA to whole length of the SFA.

Abbreviations: *CFA* common femoral artery, *XA* conventional angiography, *CT* computed tomography, *US* ultrasonography, *MRA* magnetic resonance angiography, *N/A* not available, *ASO* atherosclerosis obliterans, *LR* left-to-right direction, *AP* anterior-to-posterior direction

Lower extremity arterial embryology begins at 6-mm human embryo. The sciatic artery develops from the umbilical artery and provides the major blood supply to the lower limb bud [2, 5, 8, 12].

Between the 6–and 33-mm stage of embryo development, the iliac artery system is expanded to femoral artery system, which allows the development of lower limbs during sciatic artery regresses [6, 12]. On the 19-mm stage, multiple vascular channels known as the femoral arterial plexus are formations composed of rami femorales [5]. They develop to side-channel femoral artery rete and finally to the superficial and deep femoral arteries [2]. During development of femoral artery rete, the main arterial supply of the lower limb is the axial artery. Then, this axial artery, arising from dorsal root of the umbilical artery, is interrupted from the 19-mm embryo [2, 6, 12]. In the embryologic aspect, there is a possibility that duplicated SFA is the result of non-union of femoral artery rete. The ramus communicans superior originated from the axial artery has been proposed to act as the fusion point of the SFA duplication. The failure of formation of the ramus communicans superior results in two independent major superficial femoral artery branches [2].

In previous literature, only one report describes a case of the duplicated SFA diagnosed with US [6]. Although US examination is an easily assessable and radiation-free modality for evaluation of lower extremity arterial disorder, there are some limitations to detect duplicated vessels when the operator is unaware of anatomic variation. For example, initial US examination of other two previous case reports [7] have not necessarily revealed the duplicated SFA. Since anatomic variations of femoral artery, including the duplicated SFA, are not common and may not be easily discovered by US examination only, CTA may provide a better implemental analysis of the vascular system and its surrounding tissues [5]. In our case, the duplicated SFA was initially identified at US examination and confidently diagnosed with CTA.

In summary, we report an extremely rare case of the duplicated SFA diagnosed with the combination of US and CTA examination.

## Data Availability

All data generated or analyzed during this study are included in this published article.

## Abbreviations

SFA: Superficial femoral artery
US: Ultrasonography
CTA: Computed tomography angiography
MRA: Magnetic resonance angiography
